# Safety of ventricular tachycardia ablation under deep sedation with propofol and fentanyl

**DOI:** 10.1007/s10840-025-02081-0

**Published:** 2025-06-16

**Authors:** Vera Maslova, Sophie Lange, Tim Kannenberg, Augustin Uckermark, Julius Nebendahl, Arne Clüver, Sami Srouji, Yara Scherkus, Adrian Zaman, Fabian Moser, Derk Frank, Evgeny Lian

**Affiliations:** 1 Department of Internal Medicine III, Cardiology and Angiology, University Hospital Schleswig-Holstein, Kiel, Germany; 2 German Centre for Cardiovascular Research (DZHK), partner site North, Kiel, Germany

**Keywords:** Ventricular tachycardia, Catheter ablation, Sedation, Anaesthesia in electrophysiology, Hemodynamic stability

## Abstract

**Background:**

There is no current standard of anaesthetic management for CA of VT. Data on VT ablation under deep sedation with propofol and fentanyl are limited.

**Objective:**

The aim was to evaluate the feasibility and safety of CA of VT under deep sedation with propofol and fentanyl.

**Methods:**

Data from 134 procedures in 106 patients undergoing CA for VT under sedation with propofol and fentanyl were prospectively included. Three groups were defined and compared: group 1 (no VT induction, *n*=36); group 2 (induction of hemodynamically unstable VT, *n*=42), and group 3 (induction of hemodynamically stable VT, *n*=56).

**Results:**

Median age was 64 years, 84% were male, and 97% had structural heart disease. Group 2 had a higher proportion of patients with DCM (*p*=0.04) and severely reduced LVEF (*p*=0.024). Unipolar RF ablation was performed in 95% of procedures, bipolar in 12%, and alcohol ablation in 4%. Epicardial access was utilized in 18%. Radiation dose was higher in group 2 (*p*=0.04), while post-ablation non-inducibility was more frequently achieved in group 3 (*p*=0.045). There were no cases of profound hypotension or intubation associated with sedation. CPR was performed in seven procedures due to PEA, all in group 2 (*p*<0.001) with ROSC achieved in all cases within 3 min. No differences were observed in complication rates or hospital stay.

**Conclusion:**

CA for VT under deep sedation with propofol and fentanyl in patients with structural heart disease is feasible and safe, irrespective of VT induction, mapping, and ablation approach. Hemodynamic instability, hypotension, and desaturation can be effectively managed.

**Graphical Abstract:**

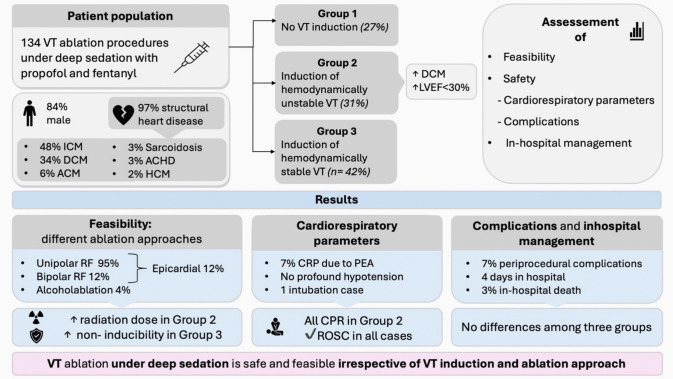

## Introduction

Catheter ablation (CA) is an established treatment of recurrent symptomatic sustained ventricular tachycardia (VT) and has shown to improve survival as well as quality of life in patients with structural heart disease (SHD) [1–5]. VT ablation is technically complex and has often long procedure duration, which requires effective pain management, airway control, and adequate patient immobilization. The majority of patients with VT have concomitant structural heart disease, either ischemic (ICM) or non-ischemic cardiomyopathy (NICM), frequently with reduced left ventricular ejection fraction (LVEF). This makes the hemodynamic management during the procedure challenging, especially during activation mapping of ongoing VT or when hemodynamically unstable VT is induced. The following VT mapping and ablation approaches are commonly used in clinical practice, according to VT induction: *substrate-based*, which does not require induction of VT, provides more hemodynamic stability during the procedure, though is less specific; *functional substrate mapping* (including conduction block lines and decremental evoked potential mapping), which is more precise and also does not require VT induction; and *activation mapping* during ongoing VT, which allows targeted ablation but may compromise the patient’s hemodynamical stability.

Anaesthetic management during CA of VT varies from minimal sedation with midazolam, deep sedation (DS) with propofol to general anaesthesia (GA) [6–9]. GA is advantageous in patients’ movement restriction, analgesia, and airway management, which are essential for safe and effective mapping and catheter stability during ablation but may impact cardiac conduction properties, such as prolonging action-potential duration and ventricular refractoriness with inhaled anaesthetics or reducing sympathetic tone with intravenous anaesthetics, potentially decreasing VT inducibility [10, 11]. Additionally, GA reduces myocardial contractility and systemic vascular resistance, which may cause acute hemodynamic decompensation and excessive hypotension during VT [11, 12]. In the case of epicardial ablation, the use of muscle relaxants could preclude identification of the phrenic nerve. The primary anaesthetic approach for VT ablation in the USA is GA [13]. The 2019 Expert Consensus Statement on Catheter Ablation of Ventricular Arrhythmias provides recommendations on anaesthesia only for idiopathic VT, advising against GA and in favour of minimal sedation in this patient group [11]. However, no information is available regarding the preferred sedation strategy for patients with SHD neither in North American nor in European guidelines [5].

DS with continuous propofol infusion is successfully implemented in the ablation of supraventricular tachycardia and atrial fibrillation (AF) [14–16]. It offers comparable efficacy and complication rates while reducing the procedure time and avoiding the GA complications [17]. Data on the safety and feasibility of VT ablation under DS is scarce.

Due to the increasing number of VT ablation procedures [18, 19] and the complexity of comorbid patients, anaesthesiologic support has become a topic of paramount importance. The aim of our study was to assess the feasibility and safety of DS with propofol and fentanyl for VT ablation in relation to VT induction and hemodynamic stability and, consequently, different mapping and ablation approaches.

## Methods

### Study population

All consecutive CA procedures for VT in our hospital in patients with and without structural heart disease, performed between January 2022 and January 2025, were prospectively enrolled. The decision to perform catheter ablation was based on current guideline recommendations [5, 11]. Both elective VT patients and patients who were admitted via the emergency room were included. Ablation of VTs under deep sedation with propofol and fentanyl is a standard practice in our department and was performed for all VT ablation procedures in the above-mentioned time interval. No procedures were performed under GA. All patients gave informed consent. The study was approved by the local ethical committee. The study complied with the Declaration of Helsinki.

### Sedation protocol and monitoring of hemodynamic parameters

Deep sedation was defined as a drug-induced depression of consciousness during which patients cannot be easily aroused but respond purposefully following repeated or painful stimulation, according to the definition of the American Society of Anesthesiologists. For the induction of sedation, a bolus of propofol of 0.8–1 mg/kg of body weight as well as 0.05 mg of fentanyl was administered followed by continuous infusion of propofol using a syringe pump. During the procedure, the infusion rate was adjusted according to clinical response (aiming for the patient immobilization and depression of consciousness) and hemodynamic parameters, such as blood pressure (BP), with a target mean arterial pressure (MAP) >65 mmHg, and peripheral oxygen saturation (SaO2), targeting >90%. Peripheral oxygen saturation was measured with a finger pulse oximeter. Oxygen was administered via a nasal cannula at an initial rate of 2 L/min with further adjustment to maintain a target SaO2 >90%. Oxygen administration via a face mask or insertion of an oropharyngeal airway tube was performed if needed. Continuous invasive blood pressure (BP) monitoring using a radial or a femoral artery line was used in all patients. Norepinephrine 10 μg/mL continuous infusion or bolus was administered via a central venous line in cases of hypotension (MAP <65 mmHg). Blood gases were not routinely analysed. An additional bolus of fentanyl intravenous (0.05–0.1 mg) was administered in case of epicardial access and prior to the beginning of the radiofrequency (RF) applications. Drugs were administered by a nurse under the supervision of a certified electrophysiologist.

### Procedure details

All patients were in a fasting state for at least 8 hours prior to the procedure. Femoral venous access with three sheaths was established in all patients and used for placement of a catheter in coronary sinus (CS), right ventricle (RV), and, in most cases, transseptal puncture. Arterial access, either via a radial or femoral line, was established in all patients for invasive BP measurement. Retrograde arterial access or epicardial access was established when needed. In the case of access to left heart chambers, heparin administration was performed prior to transseptal puncture, with activated clotting time target >300 s.

### VT induction protocol, mapping, and ablation

After catheter placement, prior to the beginning of electroanatomical mapping, the VT induction protocol was performed in all patients. The protocol consisted of a 5-beat basal train at 500 cycle length (CL), followed by up to 4 extrastimuli with a minimal coupling interval of 200 ms. Pacing was administered from the RV catheter, located in the RV apex and then in the RV outflow tract. Based on VT inducibility and patients’ hemodynamic stability during VT, the study population was divided into three different groups. *Group 1*: No VT induction. In this case, substrate mapping and substrate-based ablation with elimination of local abnormal ventricular activity (LAVA) and/or additionally ablation based on functional mapping (decremental evoked potentials mapping, conduction crowding) was performed. *Group 2*: Induction of at least one monomorphic VT, which was not hemodynamically stable, required termination either with overdrive pacing or external direct cardioversion or defibrillation. In such cases, in addition to previously described mapping and ablation strategies, pace mapping to identify the VT exit site was used, if appropriate. *Group 3*: sustained monomorphic VT (SMVT) that could be induced hemodynamically tolerated; activation mapping during ongoing VT was performed. In this scenario, targeted ablation of the slow conduction isthmus (if could be identified) was aimed for, with or without additional substrate-based techniques.

High-density mapping was performed using 3D mapping systems (CARTO Biosense Webster Inc., Diamond Bar, CA, USA; Ensite Abbott Medical; Rhythmia Boston Scientific). Low-voltage areas were defined as bipolar voltage <0.5 mV. Information about low-voltage areas in LV was collected according to the LV 17-segment model.

For ablation, a contact-force ablation catheter was used, with radiofrequency delivery power of 30–50 W. If required, alternative ablation techniques such as alcohol ablation or bipolar ablation were performed. Baseline and periprocedural characteristics were assessed and compared between the defined three groups.

### Postprocedural in-hospital management

After the procedure, patients were transferred to the general ward, intermediate care unit (IMC), or intensive care unit (ICU) based on their respiratory status, hemodynamic stability, and the need for catecholamine infusion. Data were collected on the post-ablation transfer location, reasons for ICU or IMC admission, hospitalization duration, and short-term complications (until discharge). In-hospital mortality was defined as death until discharge. These parameters were collected and compared between three groups.

### Statistical analysis

Continuous variables were reported as the median and interquartile range (25–75%) where appropriate. Categorical variables were reported as percentages. Kruskal-Wallis test was used for comparison of continuous variables with non-normal distribution among three groups and, in case of significant global effect, comparison followed using Mann-Whitney *U* test. Chi-square test was used for comparison of categorical variables. A *p*-value less than 0.05 (two tailed) was considered statistically significant. Statistical calculations were performed using the R programming language.

## Results

### Patient characteristics

**Fig. 1:**
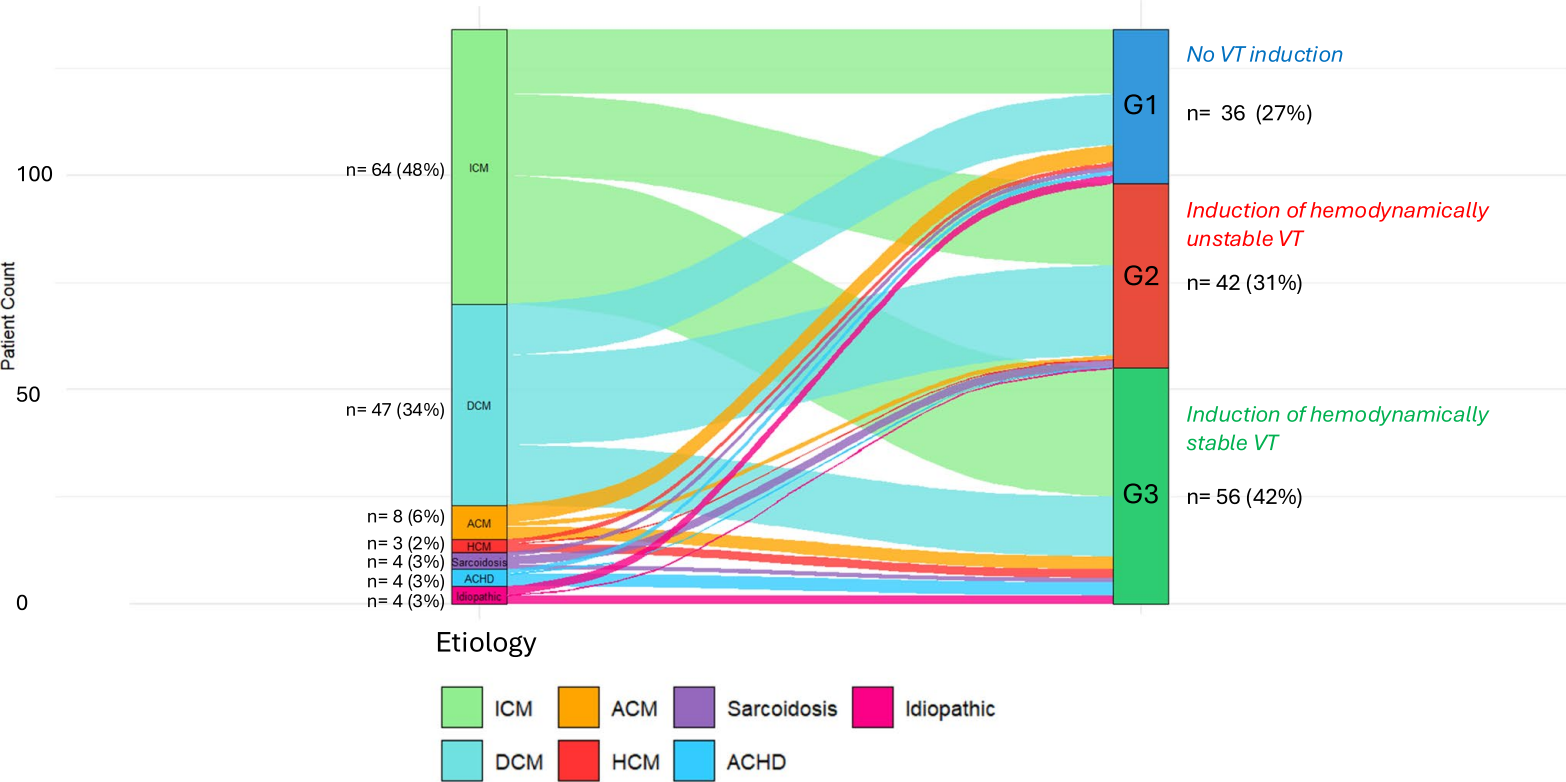
Aetiologies of structural heart disease in patient cohort and distribution according to VT induction group

A total of 134 procedures were performed in 106 patients (1.3 procedures per patient) between January 2022 and January 2025. Sixty-seven (50%) were planned elective procedures, while the same number were performed in patients admitted through our emergency room. The median age was 64 years (56–72), and in 112 (83.6%) of procedures, patients were male. The main aetiology in 64 (47.8%) procedures was ICM, in 47 (34.3%) DCM, in 8 (6%) arrhythmogenic cardiomyopathy, in 4 (3%) sarcoidosis, in 3 (2.2%) hypertrophic cardiomyopathy, and in 4 (3%) congenital heart disease in adults (ACHD) (Fig. 1). Only 4 (3%) procedures were performed for idiopathic aetiology (one VT originating from right ventricular outflow tract, two from left ventricular outflow tract, one VT from left posterior fascicle). A total of 93 (69.4%) of cases were *de novo* ablation procedures, while 41(30.6%) were repeat procedures following previous ablations either in our or an external hospital. Median LVEF was 39% (29–50), and in 9 (6.7%) of procedures, chronic lung disease was present. Previous ICD shocks were present in 46.2% of procedures, and in 16.4%, in-hospital admission was due to electrical storm. Patients’ baseline characteristics and comparisons among the three groups are listed in Table 1. Except for the higher prevalence of DCM in group 2 (33.3% vs. 50% vs. 25%, *p*=0.04) and higher prevalence of severely reduced LVEF <30% (19.4% vs. 47.6% vs. 42.9%, respectively, *p*=0.024), there was no significant difference in clinical, echocardiographic, laboratory, and ECG parameters among the three groups.

**Table 1:** Baseline characteristics of overall cohort and of three groups

	Overall (*n*= 134)	Group 1 (No VT induction) (*n*=36)	Group 2 (induction, of hemodynamically unstable VT) (*n*=42)	Group 3 (Induction of hemodynamically stable VT) (*n*=56)	*p* value
Demographics					
Age, yrs	64 (56–72)	62.5 (57–68)	66 (61–77)	62 (53–70.3)	0.18
Male	112 (83.6%)	30 (83.3%)	34 (81%)	48 (85.7%)	0.82
Comorbidities					
ICM	64 (47.8%)	15 (41.7%)	19 (45.2%)	30 (53.6%)	0.16
DCM	47(34.3%)	12 (33.3%)	21 (50%)	14 (25%)	**0.04** G1 vs. G2 *p*= 0.14 G1 vs. G3 *p*= 0.53 **G2 vs. G3** ***p*= 0.02**
ACM	8 (6%)	4 (11.1%)	1 (2.4%)	3 (5.4%)	0.29
HCM	3 (2.2%)	1 (2.8%)	0 (0%)	2 (3.6%)	0.62
Sarcoidosis	4 (3%)	1 (2.8%)	2 (4.8%)	1 (1.8%)	0.82
ACHD	4 (3%)	1 (2.8%)	0 (0%)	3 (5.4%)	0.37
Idiopathic	4 (3%)	2 (5.6%)	0 (0%)	2 (3.6%)	0.3
At least coronary one-vessel-disease	79 (59%)	18 (50%)	26 (61.9%)	35 (62.5%)	0.44
CABG	24 (17.9%)	6 (16.7%)	8 (19.1%)	10 (17.9%)	0.96
LVEF≤ 30	51 (38.1%)	7 (19.4%)	20 (47.6%)	24 (42.9%)	**0.024** **G1 vs. G2** ***p*= 0.009** **G1 vs. G3** ***p*= 0.02** G2 vs. G3 *p*= 0.23
LVEF, %	39 (29–50)	45 (35–54.3)	33.5 (28–45.8)	35 (25–50)	0.064
Atrial fibrillation	52 (38.8)	13 (36.1%)	19 (45.2%)	20 (35.7%)	0.59
Obesity*	35 (26.1%)	10 (27.8%)	11 (26.2%)	14 (25%)	0.96
CKD*	51 (38.1%)	11 (30.6%)	19 (45.2%)	21 (37.5%)	0.41
Diabetes mellitus	35 (26.1%)	9 (25%)	11 (26.2%)	15 (26.8%)	0.98
Hypertension	88 (65.7%)	24 (66.7%)	28 (66.7%)	36 (64.3%)	0.96
Chronic lung disease	9 (6.7%)	3 (8.3%)	1 (2.4%)	5 (8.9%)	0.4
PAINESD Risk Score	9 (3–12)	6 (3–9.5)	9 (5.3–12)	9 (5.8–12)	0.079
PAINESD ≤ 8 (low risk)	61 (45.5%)	21 (58.3%)	20 (47.6%)	20 (35.7%)	0.099
PAINESD 9–14 (intermediate Risk)	55 (41%)	12 (33.3%)	16 (38.1%)	27 (48.2%)	0.33
PAINESD ≥ 15 (high Risk)	18 (13.4%)	3 (8.3%)	6 (14.3%)	9 (16.1%)	0.59
Laboratory parameters					
NTproBNP, ng/l	618 (241–1499)	805.5 (196–1514.5)	577 (307.5–1518)	611.5 (293.3–1439.3)	0.95
Haemoglobin, g/dl	14 (12.9–15.2)	14.25 (13.2–15.6)	14 (12.6–14.9)	14 (13.1–14.8)	0.36
Troponin, ng/l	22.3 (12.7–39.6)	20 (14.1–51.3)	23 (12.5–39.9)	23.1 (12.9–38)	0.97
GFR, ml/min/1.73 m^2^	66 (51.3–81)	70 (55.5–87)	63 (51–76)	67 (52.8–81)	0.32
Preprocedural medication					
Beta-blocker	118 (88.1%)	32 (88.9%)	37 (88.1%)	49 (87.5%)	0.98
Amiodarone	50 (37.3%)	11 (30.6%)	16 (38.1%)	23 (41.1%)	0.59
Mexiletine	6 (4.5%)	1 (2.8%)	0 (0%)	5 (8.9%)	0.08
≥ 2 Antiarrhythmic drugs	47 (35.1%)	11 (30.6%)	12 (28.6%)	24 (42.9%)	0.27
Devices					
CRT device	42 (31.3%)	10 (27.8%)	15 (35.7%)	17 (30.4%)	0.74
ICD device	117 (87.3%)	28 (77.8%)	38 (90.5%)	51 (91.1%)	0.13
ICD shocks	54/117 (46.2%)	14/28 (50%)	17/38 (44.7%)	23/51 (45.1%)	0.9
Electrical storm	22 (16.4%)	2 (5.6%)	10 (23.8%)	10 (17.9%)	0.071
Baseline ECG					
LBBB	6/132 (4.6%)	1/36 (2.8%)	2/42 (4.8%)	3/54 (5.6%)	0.88
QRS duration	120 (100–140)	110 (90–130)	110 (100–140)	120 (100–140)	0.16
RV pacing	15/132 (11.4%)	5/36 (13.9%)	4/42 (9.5%)	6/54 (11.1%)	0.89
Biventricular pacing	35/132 (26.5%)	6/36 (16.7%)	14/42 (33.3%)	15/54 (27.8%)	0.24

*ACHD* adults with congenital heart disease, *ACM* arrhythmogenic cardiomyopathy, *CABG* coronary artery bypass graft, *CKD* chronic kidney disease, *CRT* cardiac resynchronization therapy, *DCM* dilated cardiomyopathy, *GFR* glomerular filtration rate, *HCM* hypertrophic cardiomyopathy, *ICD* implantable cardioverter-defibrillator, *ICM* ischemic cardiomyopathy, *LBBB* left bundle branch block, *LVEF* left ventricular ejection fraction, *RV* right ventricular

*CKD= GFR<60 ml/min/1.73 m^2^

*Obesity =BMI>30

Bold values indicate statistical significance (*p* <0.05)

### Periprocedural data

In 36 (26.9%) procedures, no sustained VT could be induced (group 1); in 42 (31.3%) procedures, a hemodynamically unstable ventricular arrhythmia was induced, requiring immediate termination (group 2); in 56 (41.8%), at least one hemodynamically stable SMVT was induced, and activation mapping was performed during the ongoing VT (group 3).

### Electroanatomical mapping and ablation

Transseptal access to LV was performed in most procedures (86.6%, *n*=116); in 24 (17.9%) epicardial; and in 6 (4.5%), additional retrograde transaortic accesses were performed. Electroanatomical high-density mapping with a 3D mapping system was conducted in all procedures, with CARTO used in the majority (59.7%, *n*=80), less frequently EnSite (31.3%, *n*=42), and Rhythmia (9%, *n*=12) systems. Five (3.7%) procedures were performed with Impella device support.

A total of 116 voltage maps of the LV were evaluated, with the presence of low voltage areas observed in 82 (70.7%) of them. In other cases, either only the RV voltage map was performed (14.2%, *n*=19); in one patient, LV mapping was not possible due to both mechanical mitral and aortic valves. In this patient, alcohol ablation only was performed.

No significant differences in the number and location of low voltage areas among the three groups were observed.

Radiofrequency (RF) ablation was performed in 128 (95.5%) of procedures. Of these, in 104 (81.3%) procedures, endocardial from LV, in 30 (23.4%) endocardial from RV, and in 15 (11.7%) epicardial. A total of 5 (3.7%) alcohol ablation procedures and 16 (11.9%) bipolar ablation procedures were performed. In 2 (1.5%) procedures, no ablation was performed (both were recurrent ablations with deep intramural substrate and the decision was made to perform stereotactic radiotherapy instead of RF ablation).

**Fig. 2:**
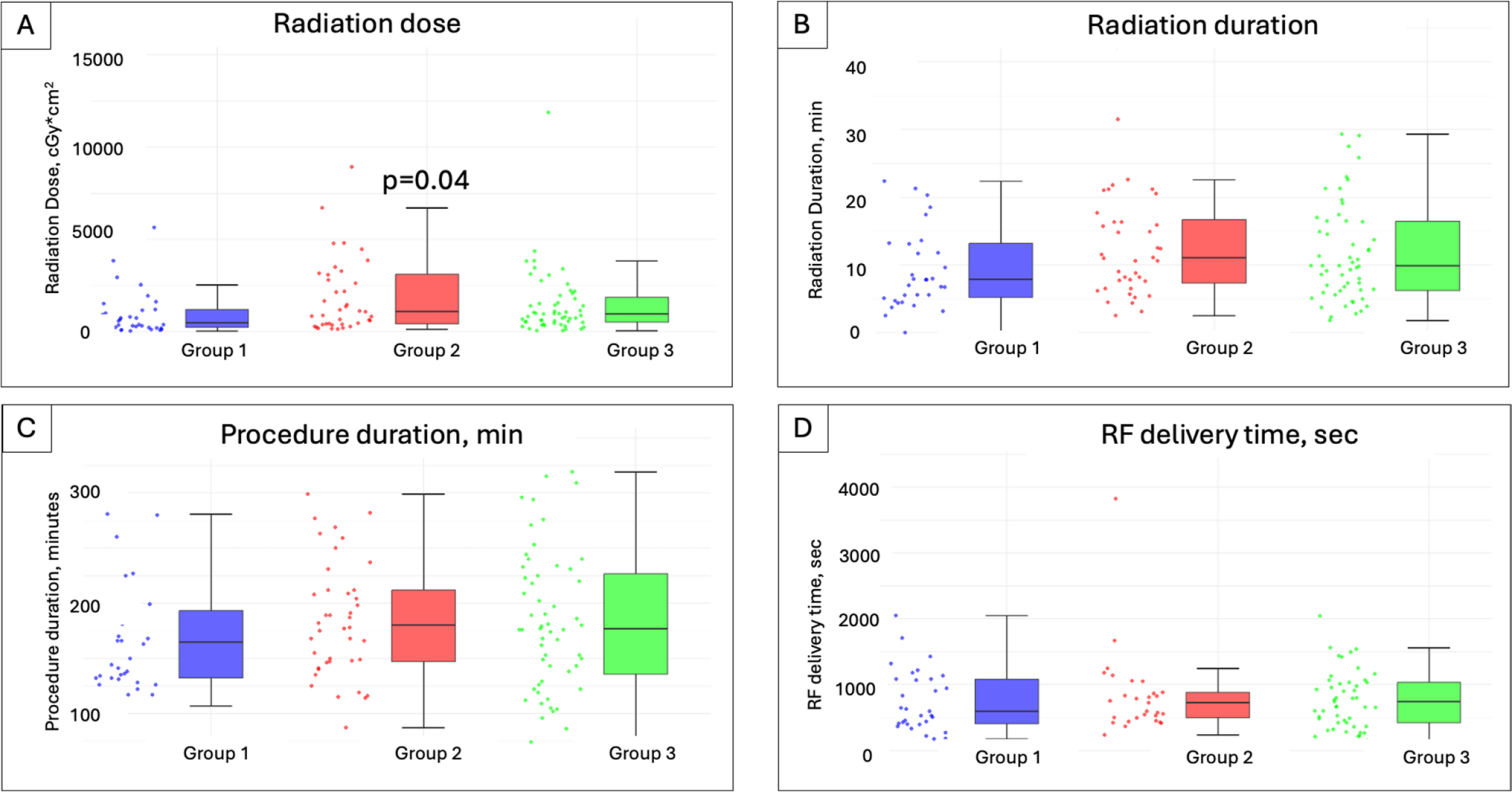
**A** Radiation dose; **B** radiation duration; **C** procedure duration; **D** RF energy delivery time across three groups. RF, radiofrequency

The median procedure duration was 176 (136.5–212) min, radiation duration 9.8 (6.2–16.3) min, radiation dose 825.8 (370.5–2038.4) cGy, and RF delivery time 665 (430–1060) s (Fig. 2).

Periprocedural parameters and comparisons among the three groups are depicted in Table 2. Except for a higher radiation dose in group 2 (484.8 vs. 1097.9 vs. 962.1 cGy, *p*=0.04), there were no significant differences. Non-inducibility of the VT could be achieved in 116 (86.5%) of cases, with a lower non-inducibility rate in group 2 (100% vs. 73.8% vs. 89.3%, *p*=0.007). When comparing non-inducibility between groups 2 and 3 (since no VT was induced in group 1 by definition), the *p*-value remained significant (*p*=0.045).

**Table 2:** Periprocedural characteristics of the study cohort and of three groups

	Overall (*n*=134)	Group 1 (no VT induction) (*n*=36)	Group 2 (induction of hemodynamically unstable VT) (*n*=42)	Group 3 (induction of hemodynamically stable VT) (*n*=56)	*p* value
LV access					
Transseptal access	116 (86.6%)	31 (86.1%)	37 (88.1%)	48 (85.7%)	0.94
Retrograde access	6 (4.5%)	1 (2.8%)	2 (4.8%)	3 (5.4%)	1.0
Epicardial access	24 (17.9%)	6 (16.7%)	9 (21.4%)	9 (16.1%)	0.77
Ablation					
Endocardial LV	104/128 (81.3%)	29/36 (80.6%)	31/38 (81.6%)	44/54 (81.5%)	0.99
Endocardial RV	30/128 (23.4%)	6/36 (16.7%)	6/38 (15.8%)	18/54 (33.3%)	0.078
Epicardial	15/128 (11.7%)	4/36 (11.1%	8/38 (21.1%)	3/54 (5.6%)	0.076
Bipolar ablation	16 (11.9%)	2 (5.6%)	4 (9.5%)	10 (17.9%)	0.2
Alcohol ablation	5 (3.7%)	0 (0%)	4 (9.5%)	1 (1.8%)	0.08
Procedure duration	176 (136.5–212)	164.5 (132–193)	180 (147.3–212)	176.5 (135.8–226.5)	0.3
Radiation duration	9.8 (6.2–16.3)	7.9 (5.2–13.2)	11.1 (7.3–16.7)	9.9 (6.3–16.5)	0.19
Radiation dose, cGy*cm²	825.8 (370.5–2038.4)	484.9 (234.4–1193.9)	1097.9 (421.2–3105.1)	962.1 (514–1871.8)	**0.04** G1 vs. G2 *p*= **0.02** G1 vs. G3 *p*= **0.05** G2 vs. G3 *p*= 0.4
RF delivery time (sec)	665 (430–1060)	600 (408–1085)	725 (498–883)	746 (425–1039)	0.9
Impella support	5 (3.7%)	0 (0%)	3 (7.1%)	2 (3.6%)	0.38
Non-inducibility after ablation	116/134 (86.5%)	36/36 (100%)	31/42 (73.8%)	50/56 (89.2%)	**0.007** **G2 vs. G3** ***p*=0.045**
Periprocedural medication and desaturation					
Midazolam admission	10 (7.5%)	3 (8.3%)	2 (4.8%)	5 (8.9%)	0.77
Midazolam dose, mg	2 (1.3–2)	2 (1.5–2)	2 (2–2)	2 (1–2)	
Fentanyl admission	132 (98.5%)	36 (100%)	41 (97.6%)	55 (98.2%)	1.0
Fentanyl dose, mg	0.1 (0.05–0.1)	0.1 (0.05–0.1)	0.1 (0.05–0.1)	0.1 (0.05–0.1)	0.39
Norepinephrine admission	94 (70.2%)	26 (72.2%)	31 (73.8)	37 (66.1%)	0.68
Desaturation (SpO2<90%)	96/131 (73.3%)	25/36 (69.4%)	31/41 (75.6%)	40/54 (74.1%)	0.82
Desaturation (SpO2<80%)	45/131 (34.4%)	10/36 (27.8%)	20/41 (48.8%)	19/54 (35.2%)	0.15
CPR	9 (6.7%)	0	9 (21.4%)	0	<0.001
LV low voltage areas in voltage map					
Presence of LV low voltage areas	82/115 (71.3%)	23/31 (74.2%)	23/36 (64%)	36/48 (75%)	0.49
Number of low voltage areas	3 (0–7)	2 (0.5–6.5)	2 (0–6)	4 (0.8–8)	0.28
Apex	54/115 (47%)	15/31 (48.4%)	16/36 (44.4%)	23/48 (47.9%)	0.94
Anterior	23/115 (20%)	4/31 (12.9%)	6/36 (16.7%)	13/48 (27.1%)	0.26
Anteroseptal	35/115 (30.4%)	8/31 (25.8%)	9/36 (25%)	18/48 (37.5%)	0.38
Anterolateral	17/115 (14.8%)	2/31 (6.5%)	6/36 (16.7%)	9/48 (18.8%)	0.3
Inferior	48/115 (41.7%)	12/31 (38.7%)	13/36 (36.1%)	23/48 (47.9%)	0.51
Inferoseptal	45/115 (39.1%)	8/31 (25.8%)	12/36 (33.3%)	25/48 (52.1%)	**0.05**
Inferolateral	35/115 (30.4%)	13/31 (41.9%)	8/36 (22.2%)	14/48 (29.2%)	0.21

*CPR* cardiopulmonary resuscitation, *LV* left ventricle, *RF* radiofrequency. Other abbreviations as in Table 1

Bold values indicate statistical significance (*p* <0.05)

### Periprocedural sedation and hemodynamic

In addition to propofol, fentanyl was administered in nearly all procedures (98.5%) with a cumulative dose of 0.1 ug. Midazolam was used in 10 (7.5%) with a cumulative dose of 2 mg (1.3–2). Norepinephrine was required in most procedures (70.2%, *n*=94), at a range starting from 0.5 ml/hour, reaching a maximum of 15 ml/hour for short periods in individual cases. As the norepinephrine infusion rate was changed throughout the procedure and adjusted to MAP, calculating the average dose is difficult. Norepinephrine was continued through the procedure if needed to manage hypotension. In cases where the blood pressure returned to target values, norepinephrine could be discontinued. In 10 patients, (five patients in group 1, three patients in group 2, and two patients in group 3, *p*=0.378), norepinephrine infusion was continued after the procedure. In most of these cases, norepinephrine could be discontinued on the same or following day.

**Fig. 3:**
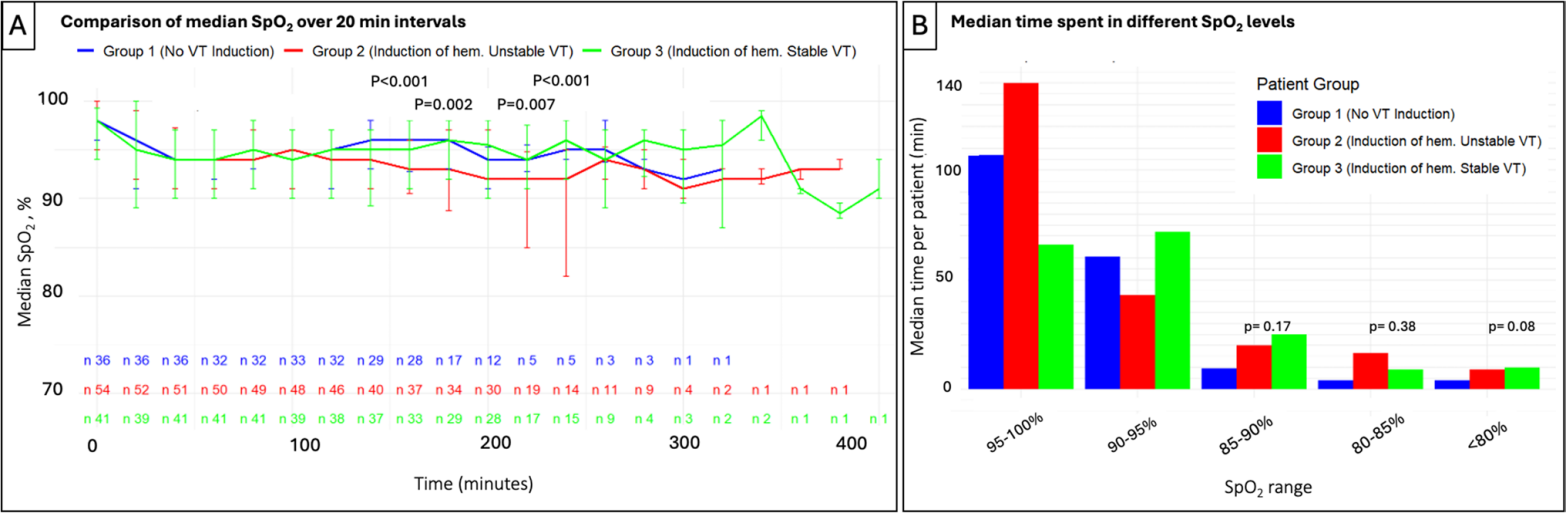
SpO_2_ levels throughout the procedure. **A** Median SpO_2_ levels of over 20-min intervals; **B** median time spent in different SpO_2_ ranges

There were no cases in which complete discontinuation of propofol infusion was necessary due to profound hypotension or desaturation. Desaturation below SpO_2_ 80% for at least 30 seconds occurred in 45 (34.6%) procedures with SpO_2_ recovery achieved in all but one case, where intubation was required (a patient with cardiogenic shock due to acute coronary artery thrombosis). There were no cases requiring periprocedural non-invasive positive pressure ventilation. The number of desaturation episodes and total duration of desaturation during the procedure did not differ among the three groups. However, SpO_2_ levels, measured over 20-min intervals, tended to be lower in group 2 (at 80, 140, 160, 180, 200, 220, 240 min, *p*<0.05 for all). A graphical representation of SpO_2_ levels during the procedure across the three groups is shown in Fig. 3.

Periprocedural cardiopulmonary resuscitation (CPR) was performed in nine cases (6.7%). In all instances, the cause of CPR was pulseless electrical activity (PEA) following an episode of hemodynamically unstable VT or VF. Manual chest compressions were required only briefly, as return of spontaneous circulation (ROSC) was achieved within 3 min in all cases, and procedures were subsequently continued. In one case, acute hemodynamic decompensation with cardiogenic shock occurred. Coronary angiogram revealed an acute thrombus in the left main coronary artery, most likely embolic, requiring thrombus aspiration.

### Complications

Serious periprocedural complications occurred in 9 procedures (6.7%) and are listed in Table 3. Pericardial effusion was observed in four cases (3%); all procedures were continued after pericardial puncture. Pericardial drainage was removed the following day in all cases. A transient ischemic attack occurred in one patient on the day of the ablation. Two patients developed third-degree atrioventricular block during the ablation, which persisted postprocedurally. Both had a septal VT origin and previously implanted ICD or CRT devices. Major bleeding occurred in one previously described patient with cardiogenic shock, who experienced retroperitoneal bleeding following Impella implantation. Complication rates did not differ significantly among the three groups.

**Table 3:** Periprocedural complications and in-hospital mortality

	Overall (*n*=134)	Group 1 (no VT induction) (*n*=36)	Group 2 (induction of hemodynamically unstable VT) (*n*=42)	Group 3 (induction of hemodynamically stable VT) (*n*=56)	*p* value
All complications	9 (6.7%)	1 (2.8%)	4 (9.5%)	4 (7.1%)	0.49
Pericardial effusion	4 (3%)	1 (2.8%)	2 (4.8%)	1 (1.8%)	0.82
Cardiogenic shock	1 (0.8%)	0	0	1 (1.8%)	1.0
Embolic events	1 (0.8%)	0 (0%)	1 (2.4%)	0 (0%)	0.58
Complete AV block	2 (1.5%)	0 (0%)	0 (0%)	2 (3.6%)	0.51
Major bleeding	1 (0.8%)	0 (0%)	1 (2.4%)	0 (0%)	0.58
In-hospital mortality	4 (3%)	0 (0%)	1 (2.4%)	3 (5.4%)	0.47

*AV*, atrioventricular

### In-hospital management

Following the majority of procedures (66.4%, *n*=89), patients were transferred to the general ward, while in 45 (33.6%) cases, monitoring in the intensive care unit (ICU) or intermediate care unit (IMC) was required for a median duration of 25 (24–48) h. The total length of hospitalization was 4 (2–9) days, and post-ablation hospital stay was 1 (1–3) nights. Four patients (3%) died after ablation before discharge: one woman from hypovolemic shock following major bleeding (previously described patient with acute coronary thrombosis), two patients with end-stage DCM from cardiogenic shock (for both, catheter ablation was performed as *ultima ratio* due to electrical storm), and one patient from electromechanical dissociation due to acute renal failure and hyperkalaemia (Table 4).

**Table 4:** In-hospital course

	Overall (*n*=134)	Group 1 (no VT induction) (*n*=36)	Group 2 (induction of hemodynamically unstable VT) (*n*=42)	Group 3 (induction, hemodynamic stability) (*n*=56)	*p* value
Transfer to ICU or IMC	45 (33.6%)	7 (19.4%)	14 (38.9%)	21 (37.5%)	0.18
Time in ICU or IMC (h)	25 (22–48)	24 (23–29.5)	27 (24–32)	24 (22–64)	0.86
Time from ablation to discharge (nights)	1 (1–3)	1 (1–2.3)	2 (1–4)	1 (1–4)	0.21
Total hospitalization duration (nights)	3 (1–8)	4 (1–8.3)	3.5 (1–8.5)	3 (1–8)	0.88
In-hospital mortality	4 (3%)	0 (0%)	1 (2.4%)	3 (5.4%)	0.47

*ICU* intensive care unit, *IMC* intermediate care unit

## Discussion

### Main findings

In this prospective single-centre study including 134 procedures, we present the results on the feasibility of propofol- and fentanyl-based deep sedation for different approaches for catheter ablation of ventricular tachycardia. To the best of our knowledge, this is the first study addressing the feasibility of the VT ablation under DS in different scenarios according to the VT inducibility and its hemodynamic tolerance. Most of the patients included had a structural heart disease and impaired LVEF and represent the typical cohort for the VT ablation.

The present data report the following main findings:

1. Catheter ablation of ventricular tachycardia under deep sedation with propofol and fentanyl appeared to be feasible across a wide range of procedures using functional mapping or activation mapping approaches, including epicardial, alcohol, and bipolar ablations as well as those requiring Impella support.
2. Hypotension and desaturation can be effectively managed without the need to discontinue sedation, even in cases of hemodynamically unstable VT or periprocedural cardiopulmonary resuscitation. However, periprocedural oxygen saturation levels tended to be lower in patients in which hemodynamically unstable VT was induced.
3. Complication rate, hospital stay duration, and in-hospital mortality did not differ based on VT induction or its hemodynamic stability.

The primary concern of using deep sedation instead of general anaesthesia for VT ablation is the challenging management of hemodynamic and airway issues. This is particularly relevant in cases of acute hemodynamic instability caused by the induction of hemodynamically unstable VT or in cases of hemodynamic decompensation resulting from prolonged VT during the activation mapping. For this reason, we defined the three previously mentioned groups according to VT induction and its hemodynamical stability. The other concerns related to DS include the following: (I) inadequate pain control during the procedure and (II) insufficient catheter stability and associated complications. The main anticipated advantages are (I) shorter procedure duration and fewer anaesthesia-related complications and (II) better inducibility of the arrhythmia. In our hospital for DS, we routinely use a combination of propofol as a sedative and fentanyl as an analgetic (as propofol has no analgetic properties); in some cases, midazolam is administered to enhance the sedative effect.

Another important aspect is that, in many countries, DS is usually managed by an anaesthesiology team rather than by electrophysiologists. Although this requires additional resources, the involvement of a dedicated anaesthesia team may make this sedation approach even safer.

### Periprocedural hemodynamic

Hemodynamic decompensation is one of the primary challenges in VT ablation with potentially devastating consequences, as many of the patients have a high burden of comorbidities, structural heart disease, and compromised LVEF [20]. Sedative agents like propofol have cardiodepressant effects, which may contribute to excessive hypotension and decompensation during the procedure [21, 22]. In our patient cohort, deep sedation with propofol and fentanyl was successfully maintained throughout the entire ablation procedure, with continuous adjustment of the propofol infusion rate according to respiratory and hemodynamic status. In cases of hypotension, norepinephrine infusion was administered effectively, and there were no cases of propofol cessation due to profound hypotension. Servatius et al. reported on VT ablations performed under sedation with propofol and fentanyl, administered under the supervision of cardiologists [23]. However, in 11.7% of cases, sedation had to be discontinued due to hypotension, despite the use of cafedrine hydrochloride/theodrenaline hydrochloride. Another study compared deep sedation with propofol and midazolam to minimal sedation with midazolam in patients undergoing catheter ablation of ventricular arrhythmias [6]. Both sedation strategies were found to be safe and feasible, with no cases of acute hemodynamic decompensation. However, unlike our study, this cohort included not only VT ablations but also procedures for premature ventricular contractions (PVC), with most patients having an idiopathic arrhythmia aetiology.

In 9 (6.7%) of procedures, acute hemodynamic decompensation (AHD) due to PEA occurred, requiring CPR and following successful ROSC within 3 min. The 6.7% AHD rate aligns with previously reported data (up to 11%) [12, 24]. In our study, these events were not attributed to high PAINESD score, which did not differ among the three groups [12]. Similarly, a recent retrospective analysis of a large VT ablation cohort found no association of PAINESD score with hemodynamic decompensation episodes [25].

### Periprocedural respiratory parameters

Sedation with propofol and fentanyl carries a potential risk of respiratory depression [26, 27]. In AF ablation procedures, sedation with propofol is well established and has been shown to be safe and effective, with a minimal number of cases requiring assisted ventilation [16, 28, 29]. In our study, although several desaturation episodes were observed, intubation and mechanical ventilation were required in only one case, which was not a direct consequence of propofol sedation, but rather of cardiogenic shock due to acute coronary thrombosis. In other cases, adjusting the propofol infusion rate and increasing oxygen delivery were sufficient for recovery of respiratory status. The tendency toward lower saturation in group 2 during the middle of the procedure may be related to the induction of hemodynamically unstable VT, leading to transient respiratory deterioration. Our findings support the safety of DS for VT ablation, even in multimorbid patients including those with chronic lung disease (present in 6.7% of procedures in our study, *n*=9), with desaturation episodes effectively managed.

### Feasibility of different ablation approaches and techniques

Adequate pain control is crucial for electrophysiology procedures, as any patient movement can lead to injury of important cardiac structures or cause a shift of the 3D map [30]. This is particularly challenging in long-lasting complex procedures such as VT ablations. In our study, pain management remained effective even during prolonged procedures, with a mean procedure duration of 176 min, as well as across various LV access approaches (transseptal, retrograde transaortic, epicardial) and different ablation strategies (endo- and epicardial, alcohol and bipolar ablation).

In 24 (17.9%) procedures, standard subxiphoid epicardial access was obtained, and in 15 (11.2%), epicardial ablation was performed. Immediately before pericardial puncture, patients received an additional dose of fentanyl for short-term analgesia, along with local anaesthesia at the puncture site. No pericardial puncture complications occurred in the cohort. The feasibility of epicardial ablation under deep sedation with propofol or other drugs such as dexmedetomidine, or a combination of remifentanil and midazolam has also been reported in previous studies [8, 23, 31]. In a significant proportion of procedures (11.9%, *n*=16), bipolar ablation was performed, with DS providing adequate sedation, pain control, and sufficient stability of both “active” and “return” ablation catheters. This aligns with recent data, which reported that 95% of bipolar VT ablation procedures were conducted under DS [32].

We observed a lower non-inducibility rate in group 2 in comparison to group 3 (*p*=0.007), which may be related to a higher prevalence of DCM patients (*p*=0.04), who are known to have complex, often intramural or epicardial substrate [33], an inability to perform the detailed mapping of the tachycardia and target the isthmus with the ablation, as well as a lower LVEF in this group. A comparison with group 1 was not performed, as these were patients, by definition, who exhibited no VT inducibility at baseline.

Data on impact of VT inducibility and its hemodynamic stability on catheter ablation outcomes remain limited. Dong et al. reported an association between non-inducibility of VT during the procedure and a higher recurrence rate at 1-year follow-up. VT was more frequently non-inducible in patients receiving deeper sedation, and a bispectral index value of <40 was associated with non-inducibility [34]. Another retrospective study reported no difference in VT inducibility but demonstrated better hemodynamic stability of VTs under conscious sedation compared to GA [10]. These findings suggest a potential benefit of lighter sedation levels for VT inducibility, hemodynamic stability, and outcomes.

The higher radiation dose in group 2 (*p*=0.04) may be explained by a higher proportion of pericardial punctures in this group (G1 16.7%, G2 21.4%, G3 16.1%, *p*=0.77). Comparable RF duration across all three groups, despite the expectation of a shorter RF duration in group 3, where hemodynamically stable VT could be induced and mapped, may be attributed to additional substrate ablation alongside isthmus ablation as well as the higher proportion of bipolar ablation in group 3.

### VT induction

Inducibility of clinical VT is important for successful mapping and definition of the critical isthmus of the VT. There are no data directly comparing GA with DS regarding VT inducibility. One retrospective study reported that 8% of patients were non-inducible under GA in comparison to previous non-invasive induction in the patient collective with scar-mediated VTs [10]. VT induction rate varies from 70% in ablations performed under GA to 87% in ablations performed under DS [23, 35]. In our study, with 97% of patients with structural heart disease, VT induction was possible in 98 (73.1%) cases, as previously mentioned, in 42 (31%) hemodynamically unstable, and in 56 (41.8%) hemodynamically stable. As in most episodes, there was no 12-lead ECG documentation of VT, it is difficult to say which proportion of induced VTs were clinical.

### Periprocedural complications

One of the primary objectives of the present study was to assess whether the periprocedural complication rate of VT ablation under sedation with propofol and fentanyl is increased and whether there are any differences based on VT induction and its hemodynamical stability. The overall periprocedural complication rate (6.7%) and in-hospital mortality rate (3%) in our cohort were comparable to previously reported data [25, 33]. No significant differences in complication rate were observed among the three groups. However, due to the small number of events, the statistical power to detect potential differences may be limited.

### Limitations

This study has an observational design, and no comparison with VT ablation under GA was performed. However, conducting a randomized trial comparing DS and GA for VT ablation does not appear to be feasible. Additionally, as nearly all patients had SHD, our findings cannot be generalized to patients with idiopathic aetiology of VT.

## Conclusion

Catheter ablation of VT under deep sedation with propofol and fentanyl in patients with structural heart disease is feasible and safe, irrespective of VT induction, mapping, and ablation approach. Hemodynamic instability, hypotension, and desaturation could be effectively managed.

## Data Availability

All relevant data are included in the article. Additional raw data are available from the corresponding author upon reasonable request.
